# Characteristics of Lightweight Concrete Based on a Synthetic Polymer Foaming Agent

**DOI:** 10.3390/ma13214979

**Published:** 2020-11-05

**Authors:** Marta Kadela, Alfred Kukiełka, Marcin Małek

**Affiliations:** 1 Building Research Institute (ITB), ul. Filtrowa 1, 00-611 Warsaw, Poland; a.kukielka@itb.pl; 2 Faculty of Civil Engineering and Geodesy, Military University of Technology, ul. Gen. Sylwestra Kaliskiego 2, 01-476 Warsaw, Poland; marcin.malek@wat.edu.pl

**Keywords:** foamed concrete, lightweight cellular concrete, pore material, synthetic foaming agent, prepared foam, mechanical properties, density, air-void structure, compressive strength, creep

## Abstract

The components of foamed concrete have a significant effect on its properties. Protein-based foamed concrete is used much more often. This study aims to assess the properties of foamed concrete with a density of around 500, 700, 800 and 1000 kg/m^3^ formed by using a synthetic polymer-based foaming agent. The distribution of pores, wet and dry density and compressive strengths were evaluated. In addition, the creep deformations of foamed concrete with different densities were measured. The difference in density of up to 170 kg/m^3^ for the highest densities was obtained. Foamed concrete with higher densities (700 and 800 kg/m^3^) showed similar characteristics of pores, which were different from those of samples with a density of 500 kg/m^3^. Compressive strength equal to 5.9 ± 0.2, 5.1 ± 0.2, 3.8 ± 0.3 and 1.4 ± 0.2 MPa was obtained for foamed concrete with a density of 500, 700, 800 and 1000 kg/m^3^, respectively. The obtained compressive strengths were higher than those found in the literature for the foamed concrete with the same densities. With increasing density, smaller creep deformations were obtained. Creep deformations were 509, 495 and 455 με for samples with densities of around 500, 700 and 1000 kg/m^3^ respectively. Deformation under long-term loading took place up to 90 days, regardless of the density of the foamed concrete.

## 1. Introduction

Due to sustainable development and the related reduction in energy consumption and CO_2_ emission [1,2,3,4,5,6], lightweight concrete, aerated concrete and foamed concrete are increasingly used [7,8]. Foamed concrete (FC) is classified as lightweight concrete with a density ranging from 280 to 1800 kg/m^3^ [9,10,11]) and with a minimum of 20% of air pore volume in the cementitious mix [12,13]. Foamed concrete was made using the pre-foaming method with physical foaming or mixing and the foaming method with chemical foaming [14,15,16]. Foamed concrete is characterized by its ability to flow, self-compact and self-level as well excellent thermal and acoustic insulation [17,18,19,20]. The compressive strength ranges from 0.21 to 10.34 MPa for a density of 300 to 1600 kg/m^3^ [8,21,22,23], which is related to its typical application [24,25,26]. In recent years, the development of foamed concrete has been observed. Amran et al. [27], Fadila et al. [28], Kang [29], Fernando et al. [30] and Portal et al. [31] produced foamed concrete for use in wall panels. Rum et al. [32] used foamed concrete as a component in a profiled composite slab. Kadela et al. [33,34,35], Drusa et al. [36,37,38], Tian et al. [39] and Lee at al. [40] used foamed concrete in a pavement or floor structure to transmit a load on a subsoil, including weak soil. Moreover, foamed concrete has been used in building foundations [20,41]. In addition, foamed concrete has the potential to become a mainstream material that uses waste material successfully as a replacement for cement or fine aggregate [42,43,44].

The components of foamed concrete (type of cement [45,46,47], water [48,49], water/binder ratio [50,51,52], additives [53,54] and admixtures [55,56,57]) have a significant effect on its properties [58,59,60]. Even foamed concrete with a compressive strength of 70 MPa can be made by the addition of polypropylene fiber and fine silica fume and used in high-performance cement [61]. However, an important role in the performance of foamed concrete is played by the foaming agent, which is used to make foam and then form enclosed pores [62]. Foaming agents can be classified into two groups, synthetic- and natural-based foaming agents [63], or five categories: rosin, synthetic, protein, composite and the latest type [64,65,66]. Some foaming agents are polymer admixtures [22,67]. In this context, foamed concrete is a special type of polymer-cement concrete [68,69,70]. Synthetic foaming agents are amphiprotic substances that are strongly hydrophilic and easily dissolve in water, yielding air bubbles [71]. The synthetic agent reduces the surface tension of the solution, which increases the stability of air bubbles. However, when adding a synthetic agent into a complex chemical environment such as concrete, the compatibility of surfactant and cement particles is critical to effectively entrain the desired air content and microstructure of concrete [71]. This may be the reason that the foamed concrete based on a protein foaming agent is used much more often [72,73,74]. In this case, air bubbles are caused by protein degradation. When the peptide linkage of large protein molecules breaks, more hydrophobic small molecules are formed. Similar to a synthetic blowing agent, this process reduces the surface tension of the solution and creates an interface to the air bubbles. The protein foam resulted in smaller isolated spherical air bubbles compared to air voids produced with synthetic foam [71]. This may result in over 80% higher compressive strength for protein-based foaming agents than for synthetic-based ones [13]. Moreover, Falliano et al. [65] obtained much higher compressive strength of foamed concretes with a density of around 400 to 850 kg/m^3^ for a protein-based foaming agent than for a synthetic-based foaming agent. This impact was far more pronounced than that observed by Panesar [71] on medium-to-high density foamed concrete. Meanwhile, Sun et al. [75] obtained around 43% and 11% higher compressive strength for synthetic-based foamed concrete compared to organic-based foamed concrete made from plant and animal glue/blood, respectively. Moreover, foamed concrete produced on the basis of a synthetic foaming agent is rarely found in the literature. Therefore, this study aims to assess the properties of hardened foamed concrete with a low density of around 500, 700, 800 and 1000 kg/m^3^ formed by using a synthetic polymer-based foaming agent. Evaluation of the foamed concrete was carried out to examine the distribution of pores and properties such as wet and dry density and compressive strength. In addition, unlike other scientists, we measured the creep deformations of foamed concrete with different densities. Assessment of behavior under long-term load is very important for structures such as roads or car park pavements, floor structures or building foundations, where foamed concrete is currently used, as mentioned above.

## 2. Materials

### 2.1. Specimen Preparation

The materials used in this study were Portland cement (Górażdże Cement S.A., Chorula, Poland), tap water and foaming agent. The industrial Portland cement was CEM I 42.5R, according to PN-EN 197-1:2012. Its chemical composition measured as per PN-EN 196-2:2013-11 (LOI—loss on ignition; IR—insoluble residue) and physical properties measured according to PN-EN 196-6:2011 are given in Table 1 and Table 2 respectively. The compressive strength of cement was determined according to PN-EN 196-1:2016-07. The phase composition was calculated according to Bogue’s formula.

**Figure 1:**
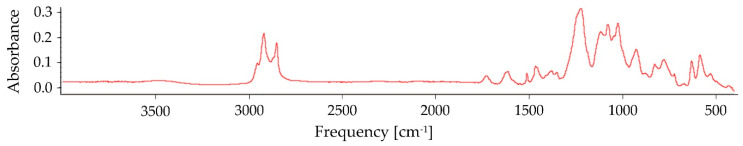
FTIR spectra of foaming agent (stearyl dimethylethylammonium methosulfate).

A commercial liquid polymer admixture with specific gravity of 1.02 g/cm^3^ was used as foaming agent. The FTIR spectra of the foaming agent are shown in Figure 1.

### 2.2. Mix Composition

The specimens of foamed concrete with four different densities were produced to investigate the effect of synthetic polymer foaming agent on the properties of foamed cementitious mix (Table 3). The densities of hardened foamed concrete were around 500, 700, 800 and 1000 kg/m^3^ with a tolerance of ±50 kg/m^3^, marked as FC500, FC700, FC800 and FC1000, respectively. The synthetic foaming agent (MEEX, Chrzanów, Poland) contents were 8.0, 6.0, 5.0 and 4.0 dm^3^ per 100 kg of cement. As reported in Table 3, the constant *w_eff_*/*c* ratio was fixed equal to 0.44 for all specimens, where *w_eff_* is water content, which includes tap water and liquid foaming agent; *c* is the cement content. This was based on the results of Jones and McCarthy [26], Xianjun et al. [76] and the authors’ earlier experiments [77,78,79].

### 2.3. Mix Production

**Figure 2:**
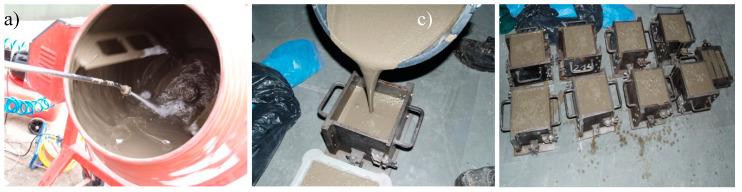
Preparation of samples: (**a**) adding foam; (**b**) pouring the mixture; (**c**) storage of samples.

The foamed concrete specimens were prepared with the pre-forming method. First, water and cement were mixed, and in the next step, the foam was added and all components were mixed (Figure 2a). In order to make a stable foam with density of 50 ± 3 kg/m^3^, the appropriate amount of liquid polymer agent was pressurized with air at approximately 5 bars using a foam generator. The foam content was established on the basis of the target dry density (considering earlier experiments); see Table 3. A tolerance of 50 kg/m^3^ was allowed on the achieved dry density. The entire manufacturing process of foamed concrete must carefully consider the densities of the mix, the foaming production rate and other factors in order to prepare high-quality foamed concrete. The key factors in producing stable foamed concrete included the pressurizing of the foaming agent at stable pressure and mixing the components in constant rotational speed.

All specimens were poured into steel molds (Figure 2b,c) and were covered with cellophane to protect against water evaporation and to ensure the best bonding conditions [65] in a curing room at 20 ± 1 °C for 24 h. Subsequently, the samples were removed from the molds and stored in a curing room at 20 ± 1 °C and 95% humidity for 14 days. After this time, the foamed concrete samples were stored under ambient conditions (at 20 ± 1 °C and 60 ± 10% humidity).

## 3. Methodology

### 3.1. General Information

The tests on hardened concrete were carried out after 28 days of curing. The material (density and distribution of pores) and mechanical properties of the concrete (compressive strength and creep deformation) were tested. Because foamed concrete is a relatively new material, there are no test standards for it in Poland or any other country [80].

### 3.2. Density

The density of the mixture was measured as per PN-EN 12350-6:2011 after pouring the mixture into the mold. The six specimens for each mix were measured.

The density of hardened foamed concrete was measured with 150 mm × 150 mm × 150 mm standard cubes as per PN-EN 12390-7:2011. The as-received specimen, i.e., naturally dried samples, was assessed. Ten measured specimens for each mix were tested.

### 3.3. Distribution of Pores

The distribution of air bubbles in the foamed concrete was analyzed using a LM microscope (OPTA-TECH STX12, Opta-Tech, Warsaw, Poland).

### 3.4. Compressive Strength

Compressive strength was measured on samples 150 mm × 150 mm × 150 mm, according to PN-EN 12390-3:2011 + AC:2012, using a compression machine MEGA 6-3000-100 (FORM+TEST, Riedlingen, Germany) having 3000 kN as max. load capacity. The loading rate was assumed as per PN-EN 772-1 + A1:2015-10 as for cellular concrete masonry units. Three samples per mix were used.

### 3.5. Creep Deformation

The creep test was carried out on beams 150 mm × 150 mm × 450 mm using a concrete creep testing machine (A-Grotex Sp. z o.o., Gliwice, Poland) in a room at 20 ± 1 °C. The load was applied continuously to the level of 1/3 of the compressive strength. For this purpose, the average compressive strength was determined on three vertically oriented base samples; see Section 3.4. Readings from measuring instruments were made at levels 1/3 and 2/3 of the specified strength. Five minutes post loading, the first strain reading was taken, recognized as an immediate strain. Then, regular readings of the total strain of the sample were carried out over a period of 1 year according to the following scheme: daily during the first week, once a week for 3 months and once a month for the rest of the period. The creep deformation after a specified time was determined by Equation (1). Four specimens per mix were used.

(1) $$\varepsilon_{c}\left(t\right)=\varepsilon_{tot}\left(t\right)-\varepsilon_{s}\left(t\right)-\varepsilon_{a}$$

where:

*ε_c_*(*t*)—creep deformation in time *t*;
*ε_tot_*(*t*)—total measured creep deformation of foamed concrete sample long-term loading in time *t*;
*ε_a_*—immediate strain determined after 5 min of obtaining 1/3 of compressive strength;
*ε_s_*(*t*)—shrinkage in time *t*.

Therefore, at the same time as the start of the long-term loading of samples, the next three samples of 150 mm × 150 mm × 450 mm were tested in the shrinkage test using Amsler’s apparatus (EMEL, Warsaw, Poland). The initial distance between the measuring points on the sample was determined. Subsequently, the samples were stored in a curing room at 20 ± 2 °C and 50 ± 3% humidity, and the distance between the measuring points on the sample was measured.

The shrinkage was determined by Equation (2). Three specimens for each density were tested.

(2) $$\varepsilon_{s}=\frac{l_{0}-l_{t,i}}{l_{0}}$$

where:

*ε_s_—*shrinkage in *i*-period;
*l*_0_, *l_t_—*initial distance between measuring points and distance after a specified curing time.

## 4. Results and Discussion

### 4.1. Density

**Figure 3:**
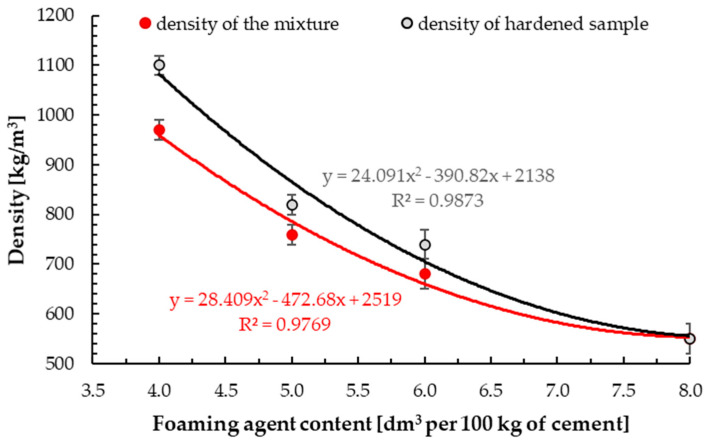
Density of the mixture and hardened samples with different content of foaming agent.

Figure 3 presents the results of average densities of the mixture and hardened sample. The density of hardened foamed concrete was 970 ± 30, 800 ± 30, 720 ± 20 and 550 ± 20 kg/m^3^ for foaming agent content of 4.0, 5.0, 6.0 and 8.0 dm^3^ per 100 kg of cement, respectively. The volume of foam commonly created air-voids and resulted in lower density [8]. The density of the mixture was 1100 ± 30, 820 ± 30, 740 ± 20 and 550 ± 20 kg/m^3^. On the basis of the achieved results, it was observed that the densities of the hardened sample from one batch of the mix were obtained with a standard deviation of ±30 kg/m^3^ and with tolerance at ±50 kg/m^3^ of the target value. This is in line with the practical method used in industrial concrete production [65,72]. The most acceptable tolerance for the density of the hardened sample is limited to ±50 kg/m^3^, which might reach a difference of up to ±100 kg/m^3^ for high density [8].

**Figure 4:**
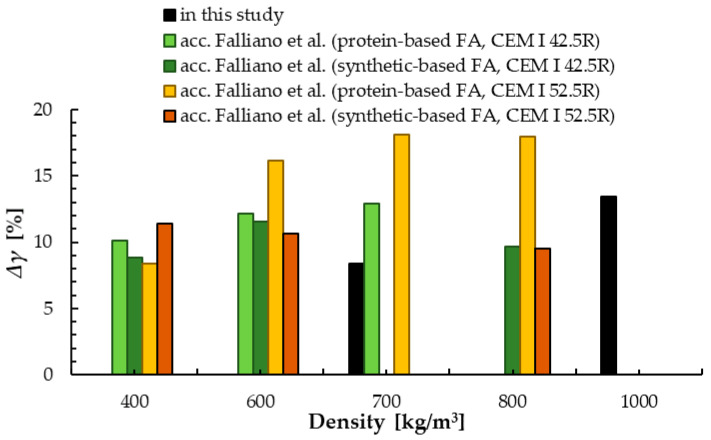
Ratio of difference between the density of the mixture and hardened foamed concrete to the density of hardened samples Δ*γ* depending on density.

The densities of the mixture and hardened samples decreased with the increase in foaming agent content [81]. These relationships were exponential. The differences between the density of the mixture and hardened sample were from 0 (this value is based on the standard deviation) to 170 kg/m^3^ depending on the density of foamed concrete. Larger differences were demonstrated for higher densities; see Figure 3. This is due to the higher air content and the lower cement material content for lower density samples. The same trend was observed by Falliano et al. [65], who obtained a difference between the density of the mixture and hardened sample of around 40 to 80 kg/m^3^ for synthetic-based foamed concrete, with density in the range of 400 to 800 kg/m^3^ and 40 to 100 kg/m^3^ for protein-based foamed concrete. Figure 4 presents the ratio of difference between density of the mixture and hardened sample to density of hardened foamed concrete. The results in this study were compared with those obtained by Falliano et al for other foaming agents (protein and synthetic) [65].

### 4.2. Distribution of Pores

**Figure 5:**
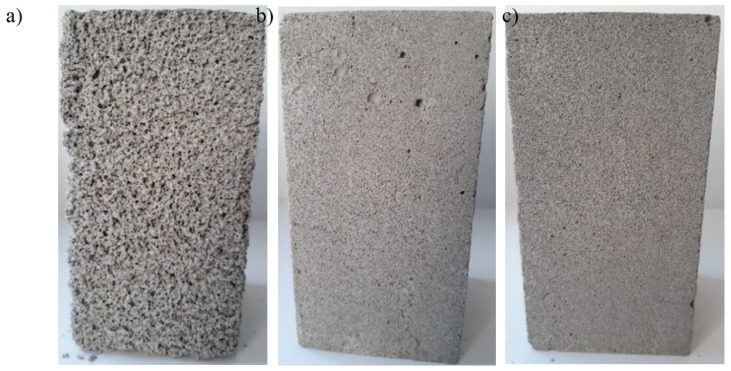
Size and distribution of pores in hardened foamed concrete samples with different densities: (**a**) 500 kg/m^3^ (FC500), (**b**) 700 kg/m^3^ (FC700), (**c**) 800 kg/m^3^ (FC800).

The decrease in density with increasing of the foaming agent content is associated with the change in the air-void structure of foamed concretes; see Figure 5. Foamed concrete is a lightweight construction material with a highly porous microstructure for all obtained densities. It can be observed that the density of foamed concrete increased with the decrease in pore size. Moreover, sometimes, larger pores were present (Figure 5b), but in general, the air-voids were quite uniformly distributed throughout the matrices [22].

**Figure 6:**
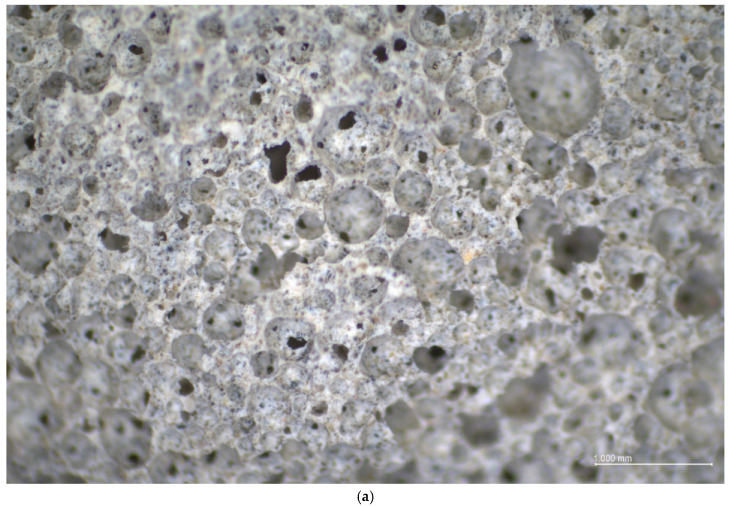

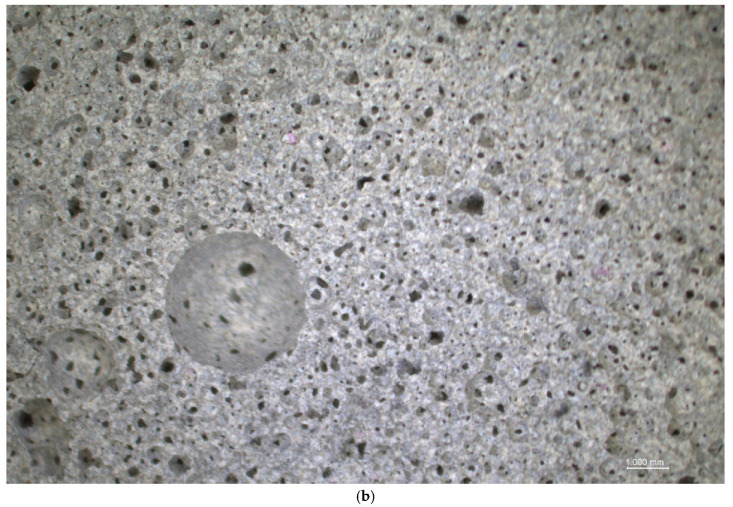
Light microscopy image of hardened foamed concrete samples with density of around (**a**) 800 kg/m^3^, (**b**) 700 kg/m^3^.

Based on Figure 5b and Figure 6a, and analogously, Figure 5c and Figure 6b (except the area with large pores), it can be observed that the samples of 700 and 800 kg/m^3^ density (FC700 and FC800, respectively) had similar characteristics of pores, such as number, area, perimeter and shape descriptors (circularity, roundness and solidity). The size of the pores ranged from 0.26 to even 3.45 mm for foamed concrete with a density of 700 kg/m^3^. This was not the case in samples with a density of 500 kg/m^3^; see Figure 5a. The number of air-voids for foamed concrete with the lowest density (FC500) was greater and the average thickness of the air bubbles wall was thinner than for the other two tested densities (FC700 and FC800). Moreover, pores of samples with density of 500 kg/m^3^ were more circular and round. The average size of pores ranged from 0.43 to 0.65 mm. The same size of pores was determined by Zhu et al. [62] for anionic surfactant as a foaming agent. Samples with a density of around 700 and 800 kg/m^3^ had smaller air-voids. This is consistent with the observation that smaller size of pores corresponds to higher density [9]. For example, Wan et al. [82] obtained air-voids between 0.010 and 0.150 mm in diameter for foamed concrete with a density of 1500 kg/m^3^; Hilal et al. [14] achieved between approximately 0.02 and 2 mm for foamed concrete with nominal densities of 1300, 1600 and 1900 kg/m^3^; and Sun et al. [75] obtained air-voids of 0.517 mm for foamed concrete with a density of 600 kg/m^3^. At the same time, more large air bubbles of up to 3.5 mm in diameter were observed in higher densities; see Figure 6. These observations were analogous to the results of foamed concrete obtained by other scientists [22,80]. In addition, according to Sun et al. [75], air-void walls in synthetic-based foamed concrete are much thicker and pore sizes are smaller and less connected than those of protein-based foamed concrete.

### 4.3. Compressive Strength

**Figure 7:**
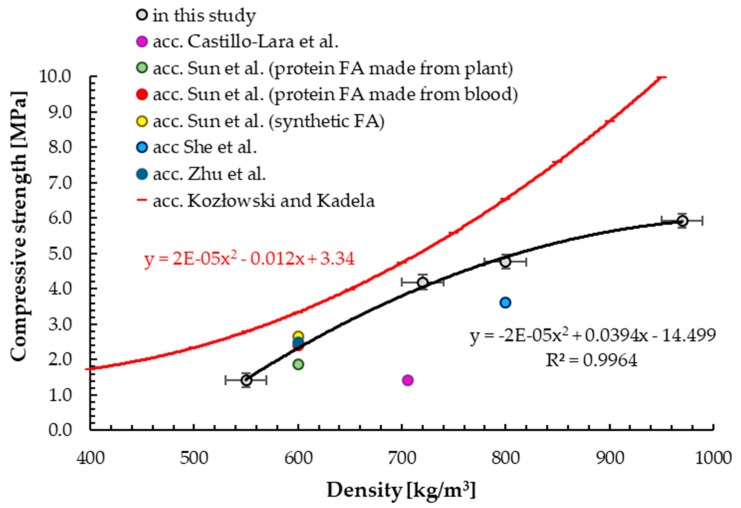
Relationship between density and compressive strength of hardened foamed concrete.

The results of compressive strength for samples with different densities are shown in Figure 7. Compressive strength is directly related with density [8]. It was observed that with the increase in density, compressive strength also increased [8,9]. With the addition of foaming agent of 4.0, 5.0, 6.0 and 8.0 dm^3^ per 100 kg of cement, the 28-day compressive strength was equal to 5.9 ± 0.2, 5.1 ± 0.2, 3.8 ± 0.3 and 1.4 ± 0.2 MPa, respectively. The addition of foaming agent decreased compressive strength because the volume of foam created pores and resulted in lower density.

The correlation between density of hardened foamed concrete and its compressive strength is exponential, which is in line with observations made by other scientists [8,59].

The results obtained in this study showed that the compressive strengths were higher than the results achieved by other scientists for the same densities, but lower than in the authors’ previous research [59], marked with the red line in Figure 7. Namely, a correlation was obtained for foamed concrete with a target density from 400 to 1400 kg/m^3^. The compressive strength obtained for higher densities influences the shape of the approximation curve. Moreover, despite the fact that the values were higher than in this study, the dispersion of the obtained results was greater. The presented diagram confirms that, depending on many factors (e.g., foam addition pressure, which was different in these tests), the obtained results may be different. This is also confirmed by the results of other scientists; e.g., Castillo-Lara et al. [83] obtained 1.42 ± 0.05 MPa for foamed concrete with density of 706 ± 8 kg/m^3^. Meanwhile, She et al. [67] achieved 3.61 MPa for foamed concrete with 800 kg/m^3^. This is due to the good structure of the foam (homogeneity of the bubbles) and its properties [80]. In addition, the synthetic foaming agent contains a stabilizer such as nanoparticles, which may accumulate at the interface of the air bubbles [67] to prevent collapse. High stability and strength of the foam will be beneficial to maintain the foam structure in the cement paste [84] and thus result in higher compressive strength. Around 43% higher compressive strength compared to plain foamed concrete (3.61 MPa) with a density of 800 kg/m^3^ was determined by using an organic stabilizer or 81% when nano-silica was used additionally [67]. Moreover, with the addition of 0.5% concentration of xanthan gum to commercial anionic surfactant foaming agents, around 48.5% higher compressive strength (3.58 MPa) compared to a reference sample of foamed concrete with a dry density of around 600 kg/m^3^ was obtained [62]. In addition, the thickness of air-void walls was doubled.

**Figure 8:**
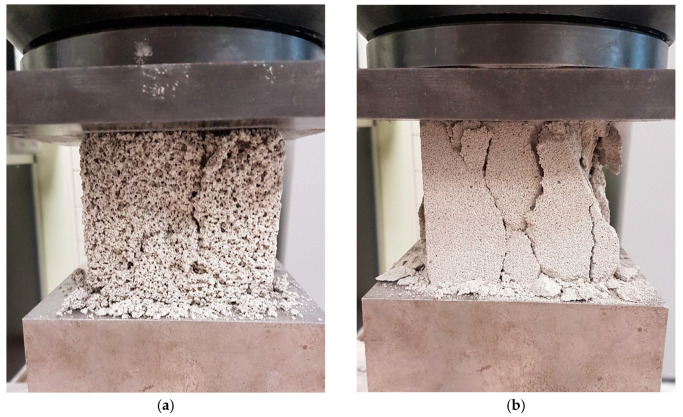
Image of destruction of foamed concrete with density of around: (**a**) 500 kg/m^3^, (**b**) 700 kg/m^3^.

Images showing the failure of foamed concrete samples with different densities are presented in Figure 8. The comparison image was obtained by Castillo-Lara et al. [83] for unreinforced foamed concrete with 706 kg/m^3^ density. Brittle behavior of foamed concrete specimens with higher density can be observed.

### 4.4. Creep Deformation

**Figure 9:**
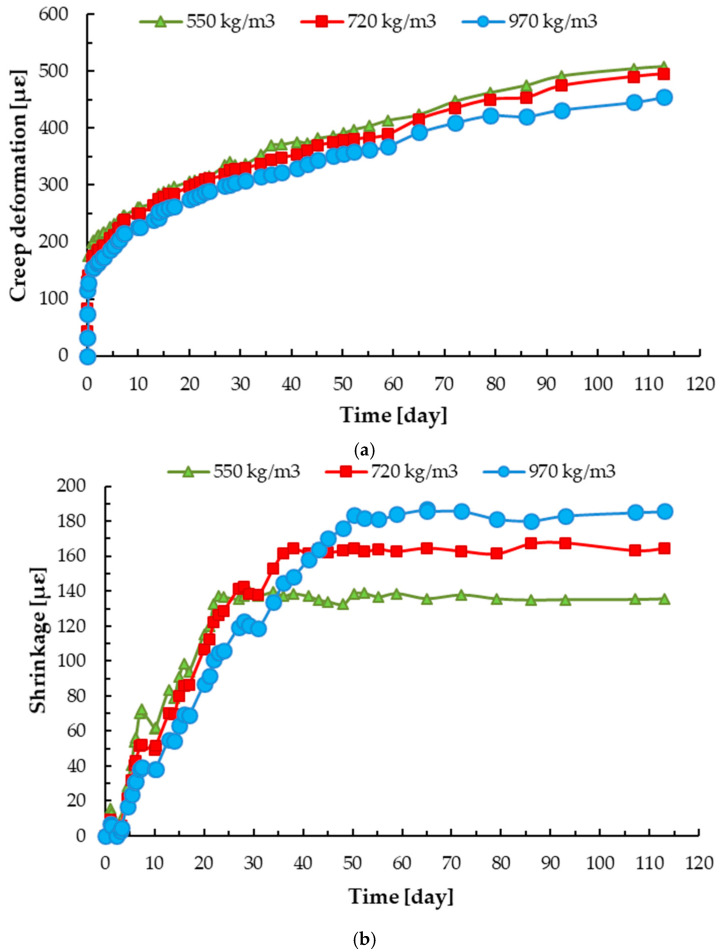
Relationship between creep deformation (**a**) and shrinkage (**b**) and time for foamed concrete with different densities.

The results of long-term loading of foamed concrete samples with different densities are shown in Figure 9a. It can be observed that for specimens with higher densities, creep deformations were smaller. Creep deformations were equal to 509, 495 and 455 με for foamed concrete with a density of around 500, 700 and 1000 kg/m^3^, respectively. Deformation under long-term loading took place up to 90 days, regardless of the density of foamed concrete. After 90 days, the differences in the values in two consecutive measurements were less than 0.2%, and therefore the creep process was considered complete. For foamed concrete with a density of around 700 and 1000 kg/m^3^, around 2.6% and 10.6% decrease compared to samples with density of 500 kg/m^3^ were obtained.

The simultaneous measurement of shrinkage strains took place in concrete for 30 to 50 days of measurement for foamed concrete with a density of around 500–1000 kg/m^3^; see Figure 9b. It can be observed that for samples with lower density, drying shrinkage was completed earlier. Moreover, for foamed concrete with a higher density, greater drying shrinkage was obtained. The presented values are relatively small, because the presented shrinkage was investigated after 28 days of hardening for foamed concrete with a density of around 500–1000 kg/m^3^. Drying shrinkage of foamed concrete was relatively high. It was found to be 4 to 10 times higher than normal concrete due to the aggregate type in the mix design, higher cement and water content and mineral admixture in foamed concrete [8]. It is reported that the drying shrinkage on day 60 was 2450 με for foamed concrete with a density of 1600 kg/m^3^, which was made of Portland cement (type I), water (*w_eff_*/*c* = 0.5) and sand [22]. For the same density of foamed concrete, but made of CEM I 52.5 Portland cement, water (*w_eff_*/*c* = 0.43) and ground granulated blast-furnace slag, Wan et al. [82] obtained a drying shrinkage of around 2000 με was observed. Sun et al. [75] determined that the drying shrinkage of foamed concrete (CEM II 52.5 Portland cement and water with *w_eff_*/*c* ratio of 0.5) with a density of around 600 kg/m^3^ on day 90 was from 2500 to 3020 με depending on the type of foaming agent used. Meanwhile, drying shrinkages of normal concrete are reported to be between 200 and 800 με [85], and for mortar, between 800 and 2000 με [86].

Drying shrinkage can be modified by selecting a suitable foaming agent type at an appropriate volume [12,87], although research carried out by Sun et al. [75] reported that drying shrinkage is slightly dependent on the origin of the foaming agent [75]. However, Wan et al. [82] determined that it depends on the hardening conditions. Meanwhile, Chindaprasirt and Rattanasak [22] demonstrated that drying shrinkage can be limited to 1520, 1430 and 1060 με with the addition of a chemical admixture.

## 5. Conclusions

The aim of the study was to assess the properties of foamed concrete made of a synthetic polymer foaming agent. This experimental work has included four different foaming agent contents (4.0, 5.0, 6.0 and 8.0 dm^3^ per 100 kg of cement), and the results obtained foamed concrete with a target density of 500, 700, 800 and 1000 kg/m^3^ with a tolerance ±50 kg/m^3^. Based on the results of this experimental investigation, the following key conclusions can be drawn:

- The density of hardened foamed concrete was 970 ± 30, 800 ± 30, 720 ± 20 and 550 ± 20 kg/m^3^ for foaming agent content of 4.0, 5.0, 6.0 and 8.0 dm^3^ per 100 kg of cement, respectively. The volume of foam commonly created air-voids and resulted in lower density.
- The differences between the density of the mixture and the density of the hardened sample were up to 170 kg/m^3^ depending on the density of foamed concrete samples; larger differences were demonstrated for higher densities. In this way, the difference between the density of the mixture and the hardened foamed concrete was obtained in order to design the mixture with the target parameters.
- Foamed concrete with a density of 700 and 800 kg/m^3^ had similar characteristics of pores, such as number, area, perimeter and shape descriptors (circularity, roundness and solidity). It can be observed that the size of pores was from 0.26 to even 3.45 mm for foamed concrete with a density of 700 kg/m^3^, while the average size of pores for foamed concrete with a density of 500 kg/m^3^ was from 0.43 to 0.65 mm.
- The number of air-voids for foamed concrete with a density of 500 kg/m^3^ was greater and the average thickness of air bubbles wall was thinner than for the other densities.
- Specimens with a density of around 700 and 800 kg/m^3^ had smaller air-voids, but more large air bubbles up to 3.5 mm in diameter were observed by higher density.
- With the addition of foaming agent of 8.0, 6.0, 5.0 and 4.0 dm^3^ per 100 kg of cement, the compressive strength was equal to 5.9 ± 0.2, 5.1 ± 0.2, 3.8 ± 0.3 and 1.4 ± 0.2 MPa, respectively. The obtained compressive strengths were higher than those found in the literature for the same densities. This was most likely related to the fact that the synthetic foaming agent contained a stabilizer foam.
- With increasing density of foamed concrete, smaller creep deformations were obtained. Creep deformations were 509, 495 and 455 με for samples with density of around 500, 700 and 1000 kg/m^3^. Deformation under long-term loading took place up to 90 days, regardless of the density of the foamed concrete.

## Figures and Tables

**Table 1:** Chemical and phase composition of cement.

**Chemical**	**SiO_2_**	**Al_2_O_3_**	**Fe_2_O_3_**	**CaO**	**MgO**	**SO_3_**	**Na_2_O**	**K_2_O**	**Cl**	**LOI**	**IR**
**Unit** (vol. %)	19.5	4.9	2.9	63.3	1.3	2.8	0.1	0.9	0.05	2.48	0.63
**Phase**	**C3S**		**C2S**			**C3A**			**C4AF**		
**Unit** (vol. %)	68		4			8			9		

**Table 2:** Physical properties of cement.

Specific Surface Area (m^2^/kg)	Specific Gravity (kg/m^3^)	Compressive Strength after Days (MPa)	
		2 Days	28 Days
3840	3060	28.0	58.0

**Table 3:** Mix proportions (1 m^3^).

Mix Symbol	Cement (kg)	*w_eff_*/*c*	Water (kg)	Foaming Agent (dm^3^ per 100 kg of cement)	Foaming Agent (dm^3^)
FC500	430	0.44	155	8.0	34.4
FC700	530		201	6.0	31.8
FC800	610		238	5.0	30.5
FC1000	700		280	4.0	28.0
